# A Comparative Analysis of the Functional Outcomes Between Retzius-Sparing and Conventional Robot-Assisted Radical Prostatectomy Using the Expanded Prostate Cancer Index Composite

**DOI:** 10.3390/cancers17243913

**Published:** 2025-12-07

**Authors:** Soichiro Shimura, Dai Koguchi, Ken-ichi Tabata, Izuru Shiba, Yutaka Shiono, Kohei Mori, Shuhei Hirano, Masaomi Ikeda, Hideyasu Tsumura, Daisuke Ishii, Kazumasa Matsumoto

**Affiliations:** 1 Department of Urology, School of Medicine, Kitasato University, Sagamihara Campus, 1-15-1 Kitasato, Sagamihara 252-0374, Kanagawa, Japan; sou-telb89@live.jp (S.S.); dai.k@med.kitasato-u.ac.jp (D.K.); junior.want.to.sleep@gmail.com (I.S.); shiono-y@med.kitasato-u.ac.jp (Y.S.); morikohei43@gmail.com (K.M.); s.hirano@med.kitasato-u.ac.jp (S.H.); ikeda.masaomi@grape.plala.or.jp (M.I.); tsumura@med.kitasato-u.ac.jp (H.T.); ishiiccf@gmail.com (D.I.); 2 Department of Urology, Kitasato Institute Hospital, Kitasato University, 5-9-1 Shirokane, Minato City 108-8642, Tokyo, Japan; ktabata@med.kitasato-u.ac.jp

**Keywords:** RS-RARP, sexual function, EPIC

## Abstract

Background/Objectives: Robot-assisted radical prostatectomy (RARP) has emerged as the predominant surgical approach globally, and various techniques have been proposed to improve functional outcomes. Despite these advances, however, functional impairment associated with surgery remains a major concern that affects patients’ quality of life. Retzius-sparing RARP (RS-RARP) has been proposed as a surgical technique to preserve the Retzius space, and increasing evidence has demonstrated its effectiveness. However, the effect of RS-RARP on other functions remains unknown. Although some reports show the positive effect of Retzius-sparing RARP (RS-RARP) on urinary incontinence, the effects on other functions remain unknown. Methods: The present study retrospectively analyzed 132 patients who underwent RARP at Kitasato University Hospital between January 2020 and July 2021 (49 and 83 patients in the RS-RARP and conventional RARP groups, respectively). We compared the functional outcomes of RS-RARP and conventional RARP (C-RARP) using the Expanded Prostate Cancer Index Composite (EPIC). In addition, sexual function was compared among patients who underwent nerve-sparing procedures. Results: Our findings revealed that, even at 12 months postoperatively, RS-RARP demonstrated significant preservation of urinary continence (*p* < 0.01) and sexual function compared to C-RARP (*p* = 0.03). No significant differences were observed in the bowel or hormonal domains. Even in patients who underwent nerve-sparing surgery, the EPIC score 12 months postoperatively was significantly higher with RS-RARP than with C-RARP (*p* < 0.01). RS-RARP tended to result in positive surgical margins more often than C-RARP (RS-RARP: 44.9%, C-RARP: 28.9%, *p* = 0.06), without affecting the rate of biochemical recurrence (RS-RARP: 14.2%, C-RARP: 15.7%, *p* = 0.81). Conclusions: Our results suggest a potential advantage of RS-RARP in preserving both urinary and sexual function.

## Simple Summary

Robot-assisted radical prostatectomy (RARP) is a common prostate cancer surgery; however, functional impairment after the procedure remains a significant concern. Retzius-sparing RARP (RS-RARP) is a technique used to improve functional outcomes. We compared the functional outcomes of RS-RARP and conventional RARP (C-RARP) using the Expanded Prostate Cancer Index Composite (EPIC). In addition, sexual function was compared among patients who underwent nerve-sparing procedures. RS-RARP demonstrated significant preservation of urinary continence and sexual function compared to C-RARP at 12 months post-surgery (*p* <0.05). Notably, among patients who underwent nerve-sparing surgery, the RS-RARP group had a significantly higher sexual summary score than the C-RARP group (*p* < 0.05). Although RS-RARP showed a slightly higher positive surgical margin rate, it did not significantly affect biochemical recurrence (*p* = 0.81). These findings suggest RS-RARP’s potential in preserving both urinary and sexual function, offering improved quality of life for patients.

## 1. Introduction

Radical prostatectomy (RP) has been the gold-standard treatment for localized and nonmetastatic prostate cancer (PCa) since the introduction of the retropubic technique in 1945 [1]. In particular, robot-assisted radical prostatectomy (RARP) can offer long-term oncological outcomes with a relatively low risk of perioperative complications compared to the laparoscopic approach [2]. Nevertheless, there remains a clinical need for improvements in functional outcomes, such as urinary continence and sexual function, following mainstream treatment. A randomized controlled trial showed a lower continence rate at 3 and 6 months after RARP by approximately 10% compared to brachytherapy for localized PCa (3 months: 86.0% vs. 98.7%, *p* < 0.05; 6 months: 90.5% vs. 98.7%, *p* < 0.05) [3]. A prospective study using the International Index of Erectile Function after RARP reported that 61% of patients had inadequate erectile function 90 days after RARP [4]. These unfavorable results highlight the need for an alternative approach to conventional RARP (C-RARP) to ensure a good quality of life after RP, with maintenance of oncological control.

One such approach is Retzius-sparing RARP (RS-RARP), initially proposed by Galfano et al. in 2010 [5]. Preserving the Retzius space contributed to the achievement of urinary continence in 90% of patients as early as 1 week after RS-RARP. Furthermore, a comparative analysis between RS-RARP and C-RARP performed by the same surgeon found that RS-RARP patients recovered to urinary continence of ≤one safety pad faster (0 to 1 safety pad, median: 44 vs. 131 days, *p* < 0.001) [6]. However, although little is known regarding the impact of RS-RARP on sexual function, anatomical analyses have revealed some proportion of neural tissue in the anterior part of the prostate. A cadaveric study found that periprostatic nerve distribution became distinct, ranging from 6.0% at the base to 11.2% at the apex, and whole-mount sections of 30 RP specimens showed variability of up to 39.9% in the anterolateral surface of the prostate [7,8]. Thus, the functional outcomes following RS-RARP are important to study, helping address the unmet needs of C-RARP. Nevertheless, data on functional outcomes following RS-RARP are scarce, especially in Asia. Given the above, the present study compared both oncological and functional outcomes between RS-RARP and C-RARP using the Expanded Prostate Cancer Index Composite (EPIC), a questionnaire querying bowel, sexual, and hormonal symptoms, to comprehensively evaluate health-related quality of life (HRQOL) [9].

## 2. Materials and Methods

### 2.1. Study Setting and Participants

The present study was conducted in accordance with the Declaration of Helsinki and was approved by the Institutional Review Board of Kitasato University (B22–114). The present study included 132 consecutive patients who underwent C- and RS-RARP between January 2020 and July 2021, consisting of 83 patients receiving C-RARP and 49 receiving RS-RARP. In this study, C-RARP was performed by 9 surgeons. A total of 29 C-RARP procedures were performed by beginner surgeons (5 surgeons) who had conducted fewer than 100 RARP procedures, or fewer than 30 RARP procedures if they had prior experience in laparoscopic radical prostatectomy. The remaining four surgeons were categorized as experienced surgeons [10,11]; RS-RARP was performed only by experienced surgeons. The procedures were performed using Da Vinci X (Intuitive, Sunnyvale, CA, USA) and Da Vinci Xi robotic systems (Intuitive, Sunnyvale, CA, USA).

### 2.2. Surgical Techniques

**Figure 1:**
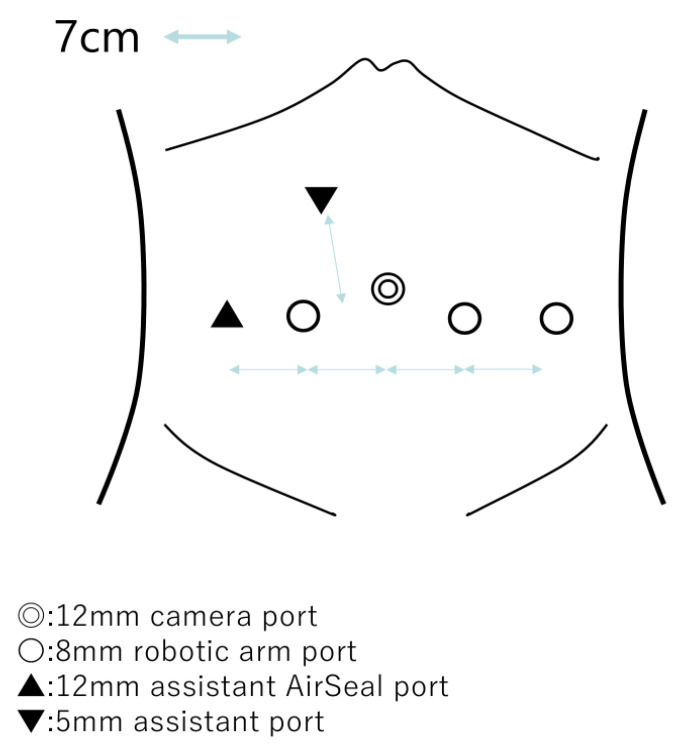
Schematic illustration of the port placement for RS-RARP.

Patients were placed in the standard 25° Trendelenburg position. Figure 1 illustrates the port layout for RS-RARP. The fourth arm was implanted on the patient’s left side.

An incision was made approximately 2 cm above the Douglas fossa. The vas deferens were dissected, and the seminal vesicle was vertically elevated towards the abdomen. The epidermis via Endo Close (Medtronic, Minneapolis, MN, USA) with 3-0 Vicryl (Ethicon, Somerville, NJ, USA) and Denonvillier’s fascia is separated by the posterolateral surface of the prostate in an antegrade direction, reaching the prostatic apex and maintaining a completely intrafascial plane. Subsequently, the lateral surface of the prostate was dissected, and bladder neck preservation was performed. To evert the mucosa of the bladder and easily identify the bladder neck orifice for anastomosis, four short cardinal stitches were positioned. The ventral surface of the prostate was then isolated, and the prostate was freed. 3-0V-Loc (Medtronic) was used for vesicourethral anastomosis, and the peritoneum was sutured. The procedure followed the protocol described by Galfano et al. [5].

At our institution, the indications for RS-RARP are as follows: prostate size ≤ 60 mL (RS-RARP was performed upon strong patient preference, even if the prostate size exceeded 60 mL), absence of median lobe hyperplasia, and no evidence of carcinoma in the apex of the prostate as determined by magnetic resonance imaging (MRI) or a prostate biopsy. A nerve-sparing procedure was performed on the side of the prostate when the prostate biopsy was not positive for PCa, or when PCa was present only in the transitional zone.

### 2.3. Analytical Data and Outcome Measures

The following data on patient characteristics were collected from the patients’ medical charts: age, body mass index (BMI), preoperative serum prostate-specific antigen (PSA) levels, preoperative Gleason score, clinical T stage, preoperative prostate volume, presence of a nerve-sparing technique, console time, intraoperative blood loss, postoperative pathological findings including T stage, Gleason score, positive surgical margin rate, and biochemical recurrence rate.

**Figure 2:**
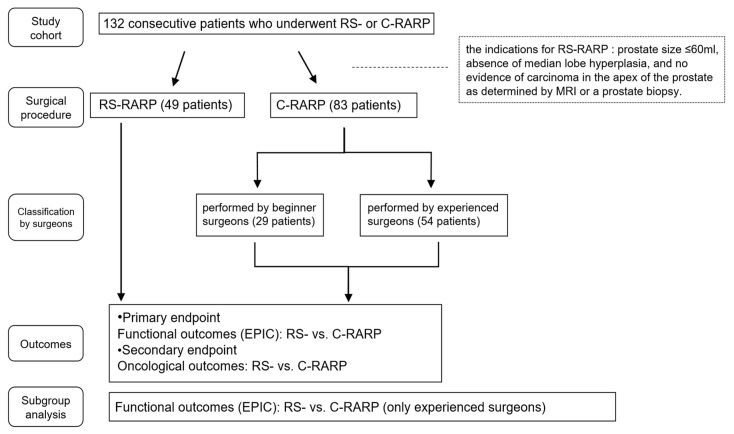
Flow chart of the study design. Abbreviations: RARP, robot-assisted radical prostatectomy; RS, Retzius-sparing; C, conventional; EPIC, Expanded Prostate Cancer Index Composite.

The primary endpoint of this study was to assess the differences in the HRQOL between the RS- and C-RARP groups. HRQOL between RS-RARP and C-RARP was assessed using the EPIC, a validated 50-item questionnaire that evaluates the urinary, bowel, sexual, and hormonal functional domains. Each domain is scored from 0 to 100 points, with higher scores indicating a better quality of life [9]. We compared the HRQOL of the RS-RARP and C-RARP preoperatively and at 1, 3, 6, and 12 months postoperatively. In addition, we compared the HRQOL between the groups that underwent nerve-sparing in RS-RARP and C-RARP procedures. We also assessed the subscale domains that subdivided the four HRQOL items included in the EPIC assessment method [9]. The subscale domains for urinary features were divided into urinary frequency, urinary bother, incontinence, and irritative/obstructive; those for bowel features were divided into bowel function and bowel bother; those for sexual features were divided into sexual function and sexual bother; and those for hormonal features were divided into hormonal function and hormonal bother. We used the mean EPIC scores for each domain for statistical analyses. The secondary endpoint of this study was to compare RS-RARP and C-RARP with respect to biochemical recurrence after surgery to evaluate the oncological outcomes of RS-RARP (Figure 2).

We evaluated the biochemical recurrence rate by dividing the prostate into six regions based on positive margins: anterior-apex, posterior-apex, anterior-middle, posterior-middle, posterolateral, and bladder neck [12]. Biochemical recurrence was defined as postoperative PSA levels of ≥0.2 ng/mL, as proposed by the American Urological Association (AUA) [13]. To minimize the influence of less experienced surgeons, we analyzed functional outcomes assessed by the EPIC questionnaire, comparing RS-RARP with C-RARP cases performed by experienced surgeons only (54 cases), excluding those performed by beginner surgeons (29 cases).

### 2.4. Statistical Analyses

Comparisons between the two RARP groups were performed using the chi-squared test (or Fisher’s exact test, if appropriate) for categorical variables and the *t*-test for continuous variables. All statistical analyses were performed using the Stata software program, version 16 for Windows (Stata, Chicago, IL, USA). All *P* values were two-sided, and *p* <0.05 was considered to indicate significance.

## 3. Results

Table 1 summarizes the baseline characteristics of the 132 patients in terms of their preoperative demographics and perioperative surgical findings. There were no marked differences between RS-RARP and C-RARP in factors related to D’Amico risk classification, such as the PSA levels (median: 7.6 ng/mL vs. 8.5 ng/mL, *p* = 0.94), Gleason score (≥ 8: 36.6% vs. 41.0%, *p* = 0.61), and preoperative T stage (≥T3: 20.4% vs. 26.5%, *p* = 0.77). The preoperative prostate volume was also comparable between RS-RARP and C-RARP (median: 30.6 mL vs. 36.0 mL, *p* = 0.11). The rate of nerve-sparing procedures was equivalent between RS-RARP and C-RARP (40.8% vs. 39.8%, *p* = 0.91). Pathological seminal vesicle invasion was observed in three patients (6.1%) receiving RS-RARP and six (7.2%) receiving C-RARP, and bladder neck invasion in one patient (1.2%) receiving C-RARP. Although RS-RARP was associated with a greater number of positive surgical margins and C-RARP had fewer (44.9% vs. 28.9%, *p* = 0.06), no significant difference was observed in terms of biochemical recurrence (14.2% vs. 15.7%, *p* = 0.81). Within 1 year after surgery, one patient (2.0%) in the RS-RARP group and seven patients (8.4%) in the C-RARP group initiated hormonal therapy, while five patients (9.8%) in the RS-RARP group and five patients (6.0%) in the C-RARP group underwent salvage pelvic floor radiotherapy. The number of patients who underwent nerve-sparing surgery and received hormonal therapy less than 1 year after surgery was zero (0%) and two (2.4%) in the RS-RARP and C-RARP groups, respectively.

Table 2 lists the 46 patients with positive surgical margins categorized by the procedure and location of the margin. For RS-RARP, the most common site of positive surgical margins was the anterior-middle (14.3%), and for C-RARP, the most common site was the anterior-apex (8.4%). We changed the surgical technique to remove more of the detrusor apron anterior to the prostate starting in the second year after the introduction of RS-RARP. As a result of the change in the surgical method, the positive surgical margin rate improved from 53.3% (16 out of 30 patients) in the first year of introduction to 31.6% (6 out of 19 patients) in the second year (*p* = 0.14). The positive surgical margin rates for RS-RARP performed in the second year and overall C-RARP were similar (RS-RARP performed in the second year: 31.6%, C-RARP: 28.9%, *p* = 0.82). Regarding the anterior site, the positive surgical margin rate was 30.0% (9 out of 30 patients) in the first year and 21.1% (4 out of 19 patients) in the second year, thus indicating an approximately 10% decrease (*p* = 0.49).

**Figure 3:**
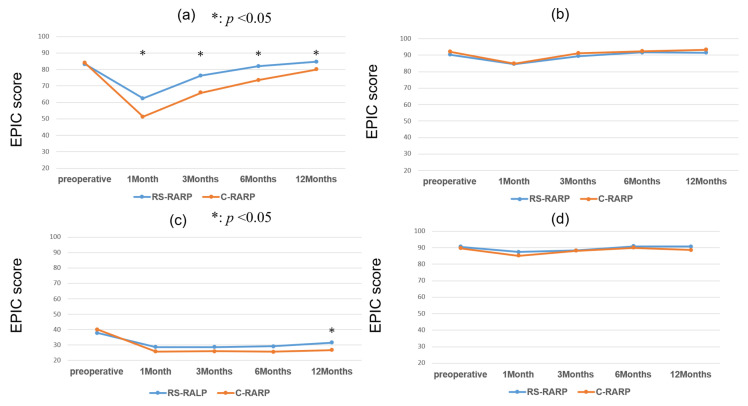
EPIC HRQOL domain summary scores: (**a**) urinary summary; (**b**) bowel summary; (**c**) sexual summary; (**d**) hormonal summary.

Figure 3 presents the HRQOL domain summary scores for EPIC. In urinary summary scores (Figure 3a), the difference in scores between RS-RARP and C-RARP gradually decreased from 11 points at 1 month after surgery to 5 points at 1 year after surgery (3 months: 11 points, 6 months: 8 points). However, the RS-RARP score was significantly higher than the C-RARP from 1 to 12 months after surgery (*p* < 0.01). Comparing preoperative and postoperative scores, the difference in urinary summary scores for RS-RARP improved until it was no longer statistically significant after 6 months, whereas the difference in urinary summary scores for C-RARP improved until it was no longer significant at 12 months. Sexual summary scores were also significantly higher in the RS-RARP group than in the C-RARP group at 12 months postoperatively (*p* < 0.03) (Figure 3c). For both RS-RARP and C-RARP, the postoperative sexual summary scores remained significantly lower than the preoperative scores even 12 months after surgery.

**Figure 4:**
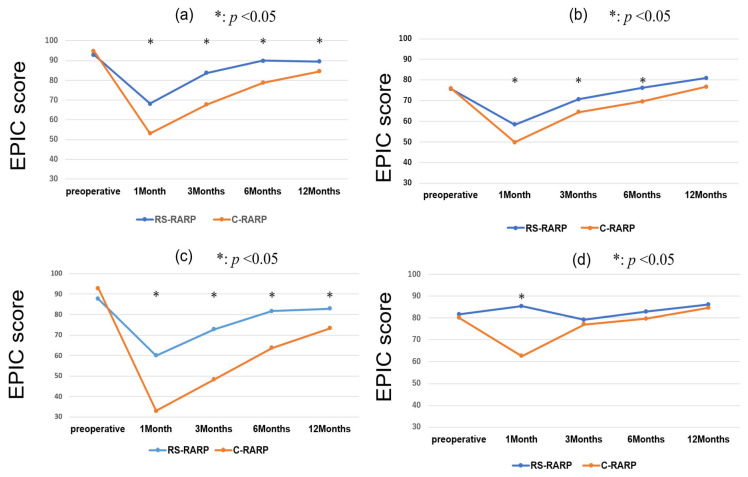
EPIC HRQOL urinary domain subscale scores: (**a**) urinary frequency; (**b**) urinary bother; (**c**) incontinence; (**d**) irritative/obstructive.

Figure 4 shows the urinary and bowel HRQOL subscale scores for the EPIC. In the urinary section, the incontinence score (Figure 4c) was consistently significantly higher in RS-RARP than in C-RARP postoperatively (from 1 to 12 months postoperatively, *p* < 0.01). The significant difference in incontinence scores between preoperative and postoperative periods in the RS-RARP disappeared 12 months after surgery, but the significant difference remained in C-RARP even 12 months after surgery. As with incontinence, RS-RARP scores were significantly higher than C-RARP scores in terms of urinary frequency (Figure 4a) from 1 to 12 months after surgery. For urinary bother, RS-RARP scores were significantly higher than C-RARP scores from 1 to 6 months after surgery, but there was no significant difference at 12 months after surgery. Regarding bowel bother (Figure S1b), the C-RARP scores were significantly higher than the RS-RARP scores at 12 months postoperatively (*p* < 0.01), but the C-RARP scores were already significantly higher preoperatively (*p* < 0.01). The disappearance of any significant difference in the bowel bother scores in comparison to the preoperative scores was observed for both RS-RARP and C-RARP.

Figure S1b shows the sexual and hormonal HRQOL domain subscales scores for EPIC. With regard to the sexual function (Figure S2a), a significant difference between preoperative and postoperative results remained for RS-RARP even at 12 months after surgery, but RS-RARP was consistently significantly higher than C-RARP from 1 to 12 months after surgery (*p* < 0.05). Sexual bother scores (Figure S2b) were significantly higher in RS-RARP than in C-RARP at 6 months and 12 months postoperatively (*p* < 0.05). For both RS-RARP and C-RARP, significant differences in sexual bother from the preoperative to postoperative periods were recovered one month after surgery.

**Figure 5:**
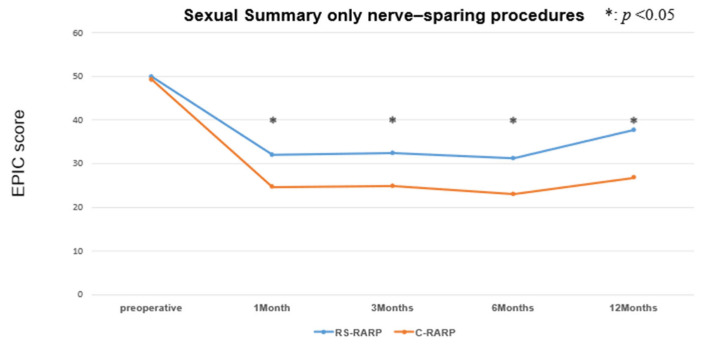
EPIC sexual summary score (only for nerve-sparing procedures).

Table S1 shows the characteristics of the patients who underwent nerve-sparing surgery. RS-RARP patients had a 2.4 kg/m^2^ lower BMI than C-RARP patients (median: 22.5 kg/m^2^ vs. 24.9 kg/m^2^, *p* = 0.003). There was no marked difference between RS-RARP and C-RARP in the proportion of patients who received unilateral or bilateral nerve-sparing (both: unilateral; 85%, bilateral; 15%). The sexual summary score was consistently significantly higher with RS-RARP than with C-RARP from 1 to 12 months after surgery (*p* < 0.01) (Figure 5). At 12 months after surgery, the sexual function score in the RS-RARP group was significantly lower than the preoperative score, but recovered to 76% (mean, 12 months after surgery: 37.7 points, preoperative: 49.9 points) of the preoperative score. Twelve months after surgery, the sexual function score in the C-RARP group was 55% (mean, 12 months after surgery: 26.8 points, preoperative: 49.2) of the preoperative score.

In the subgroup analyses of patients with C-RARP performed by experienced surgeons, Figures S2 and S3 present the domain summary EPIC scores and sexual summary scores only for nerve-sparing cases. Figure S3 presents the domain summary EPIC scores of 54 patients who were operated on by experienced surgeons. Urinary summary scores (Figure S3a) were significantly higher with RS-RARP than with C-RARP (experienced surgeons) from 1 to 12 months postoperatively (*p* < 0.05). Bowel summary scores (Figure S3b) were significantly higher with C-RARP (experienced surgeons) than with RS-RARP at 1 and 12 months postoperatively (*p* = 0.04, *p* < 0.01, respectively). Sexual summary scores (Figure S3c) were similar between RS-RARP and C-RARP (experienced surgeons) at 1, 3, and 6 months postoperatively, but scores were significantly higher with RS-RARP than with C-RARP at 12 months (*p* < 0.05). In addition, Figure S4 shows the results among patients who underwent nerve-sparing procedures. When C-RARP was limited to cases performed by experienced surgeons, the findings were consistent with those observed in the overall cohort; the sexual summary score remained significantly higher with RS-RARP than with C-RARP from 1 to 12 months after surgery (*p* <0.01).

## 4. Discussion

The present study suggests a potential advantage of RS-RARP over C-RARP with regard to not only urinary continence but also sexual function, as assessed via the EPIC. Notably, better sexual function following RS-RARP compared to C-RARP remained even after focusing our analysis on patients who received nerve-sparing RARP. In addition, we conducted subgroup analyses to validate differences in these functional outcomes between RS- and C-RARP in the cases performed by only experienced surgeons and found that urinary and sexual summary scores were higher with RS-RARP than with C-RARP.

Data regarding the association between RS-RARP and preserved postoperative sexual function are limited. However, a large retrospective study by Galfano et al. reported that 40% of patients who underwent complete intrafascial nerve-sparing surgery were able to have sexual intercourse within 1 month after surgery [14]. One such mechanism underlying the favorable postoperative sexual function following RS-RARP might involve the unique surgical approach of RS-RARP, which preserves the Retzius space and pelvic floor muscle, thereby facilitating preservation of the accessory pudendal arteries (APAs) and dorsal vascular complex (DVC). The APAs, which are located along the lateral aspect of the prostate superficial to the endopelvic fascia or penetrate through the levator ani behind the endopelvic fascia in close proximity to the apex of the prostate, supply blood to the corpus cavernosum [15]. Therefore, it is reasonably accepted that its transection is associated with postoperative erectile dysfunction [16]. In this scenario, the surgical approach of RS-RARP potentially reduces the thermal and mechanical damage of the APAs by preserving the Retzius space and pelvic floor muscle compared to C-RARP. Likewise, regarding the DVC, the unique approach of RS-RARP also maintains a large amount of the DVC, which contains not only nerves but also small arteries and external urethral sphincter fibers [17,18]. Regarding nerve pathways, it has been reported that periprostatic nerve fibers are distributed around the prostate on all sides, with a significant percentage of these fibers present in the anterior and anterolateral sectors [19]. Therefore, RS-RARP, which dissects the bladder neck from the posterior midline, can preserve the neurovascular bundle plate without injury and would thus be considered to better preserve erectile function than C-RARP. The preservation of these complexes, which critically contribute to erectile ability, can explain the better EPIC score concerning sexual function for RS-RARP compared to C-RARP, even among patients who underwent nerve-sparing procedures, in the present study. For these reasons, RS-RARP may be worth especially considering for patients who wish to preserve their sexual function.

Our study also demonstrated earlier recovery of the EPIC score in urinary continence for RS-RARP than for C-RARP. Given the specific surgical approach of RS-RARP, increasing evidence of its superiority in continence can be theoretically understood [20]. Moreover, it should be noted that such superiority of urinary continence for RS-RARP was maintained one year after surgery compared to C-RARP. This difference at postoperative one year was also maintained even among cases with nerve-sparing procedures, indicating another background to the nerve-sparing procedure. Intensive research has already revealed multiple factors associated with better urinary continence in patients with C-RARP, and specific data on RS-RARP are scarce [21]; however, a recent study conducted in Japan uniquely demonstrated the mechanism underlying the superiority in urinary continence for RS-RARP using dynamic magnetic resonance imaging [22]. This retrospective study performed imaging tests before and after the surgeries and found that the anterior bladder wall was attached to a higher position after RS-RARP than after C-RARP. Subsequently, RS-RARP could more effectively take advantage of the bladder when patients require an increasing degree of urethral closure during abdominal pressure. In fact, a meta-analysis comparing urinary incontinence with use of 0–1 pads/day 12 months after RS-RARP and C-RARP showed that more patients with RS-RARP had urinary incontinence controlled with 0–1 pads/day [23].

One issue with this surgical approach is the high rate of positive margins. Galfano et al. reported an improvement in positive margin rates between the initial 100 cases and subsequent cases, attributing this to technical refinement [14]. Meta-analyses have shown that RS-RARP tends to have a higher rate of positive resection margins than C-RARP, with a higher rate of positive margins ventral to the prostate as the site [24,25,26]. In this study, the area with the most positive surgical margins in RS-RARP was also in the anterior middle region. Although the positive surgical margin rate for RS-RARP tended to be higher than that for C-RARP, the biochemical recurrence rate was similar. It has been suggested that biochemical recurrence may differ depending on the location of positive margins, and positive margins in the posterolateral have been reported to be an independent risk factor for biochemical recurrence [27]. Prostate cancer is known to invade and metastasize along the nerves, and the abundant nerve tissue in the posterolateral wall is thought to be responsible for the increased risk due to the biochemical recurrence of residual cancerous tissue at the same site [12]. In the present study, RS-RARP had a higher number of positive anterior sites than C-RARP, owing to the characteristics of the procedure. It is possible that the positive anterior sites may have contributed to the high rate of positive margins but did not lead to biochemical recurrence.

Several limitations associated with the present study warrant mention. First, this study was a retrospective analysis of the clinical records. Second, the indication for RS-RARP was left to the discretion of the surgeon, and strict criteria were not established. Third, the group of surgeons performing C-RARP included beginners. Because RS-RARP provides a narrower working space than C-RARP, all beginner surgeons at our institution perform only C-RARP. This institutional policy may have influenced the study results. In this study, RS-RARP was compared to C-RARP performed by experienced surgeons after excluding procedures performed by beginner surgeons. However, because RS-RARP procedures were performed exclusively by experienced surgeons, it was not possible to compare RS-RARP with C-RARP performed by beginner surgeons. Fourth, this study only included Japanese patients. Japanese people reportedly have a higher frequency of erections than Westerners but a lower frequency of sexual activity. This result may have affected the scores for the sexual components of the EPIC [28,29,30]. Finally, long-term follow-up of RS-RARP patients is necessary to ensure that there is no significant compromise in long-term oncologic efficacy. Despite these limitations, RS-RARP has shown potential advantages over C-RARP, not only in terms of urinary continence but also in preserving sexual function.

## 5. Conclusions

RS-RARP may be more effective than C-RARP in terms of patient-reported outcomes, particularly for early recovery from urinary incontinence and preservation of urinary and sexual function at one year postoperatively. Notably, the sexual function was better preserved with RS-RARP than with C-RARP, even among patients undergoing nerve-sparing procedures. RS-RARP may therefore be a valuable option, especially for patients prioritizing their sexual function. Further large-scale studies are warranted to clarify the clinical significance of these findings.

## Figures and Tables

**Table 1:** Summary of patient and disease characteristics and outcomes.

Variables	RS-RARP (*n* = 49)	C-RARP (*n* = 83)	*p*
Preoperative data			
Age, median ± SD	72 ± 5.8	68 ± 5.5	0.003
BMI (kg/m^2^), median ± SD	22.6 ± 2.8	24.4 ± 3.3	0.03
PSA (ng/mL), median ± SD	7.6 ± 7.4	8.5 ± 7.9	0.94
Prostate volume(ml), median (IQR)	30.6 (22.9–45.4)	36.0 (26–53.7)	0.11
Gleason score, *n* (%)			
6	10 (20.4)	16 (19.3)	0.68
7	21 (42.9)	33 (39.8)	
8	12 (24.4)	17 (20.5)	
≥9	6 (12.2)	17 (20.5)	
T stage, *n* (%)			
≤2	44 (89.8)	73 (88.0)	0.77
T3	5 (10.2)	10 (12.0)	
Nerve-sparing procedure, *n* (%)			
Yes	20 (40.8)	33 (39.8)	0.91
No	29 (59.2)	50 (60.2)	
Postoperative data			
Console time (minutes), median ± SD	188 ± 48.2	205 ± 72.6	0.03
Blood loss (ml), median ± SD	200 ± 255	263 ± 314	0.07
T stage, *n* (%)			
≤T2	39 (79.6)	61 (73.5)	0.62
≥T3	10 (20.4)	22 (26.5)	
Gleason score, *n* (%)			
6	7 (14.3)	7 (8.4)	0.21
7	31 (63.3)	49 (59.0)	
8	3 (6.1)	15 (18.1)	
≥9	8 (16.3)	11 (13.3)	
Positive surgical margin, *n* (%)	22 (44.9)	24 (28.9)	0.06
Biochemical recurrence, *n* (%)	7 (14.2)	13 (15.7)	0.81

Abbreviations: SD, standard deviation; IQR, interquartile range; RARP, robot-assisted radical prostatectomy; RS, Retzius-sparing; C, conventional; BMI, body mass index; PSA, prostate-specific antigen.

**Table 2:** Number and location of sites of positive surgical margins.

		Anterior			Posterior		
Procedure	Total	Apex	Middle	Bladder neck	Apex	Middle	Posterolateral
RS-RARP (*n* = 49)	22 (44.9)	5 (10.2)	7 (14.3)	1 (2.0)	4 (8.2)	2 (4.1)	3 (6.1)
C-RARP (*n* = 83)	24 (28.9)	7 (8.4)	4 (4.8)	4 (4.8)	3 (3.6)	2 (2.4)	4 (4.8)

RS-RARP, Retzius-sparing robot-assisted radical prostatectomy; C-RARP, conventional robot-assisted radical prostatectomy.

## Data Availability

The datasets used and/or analyzed during the current study are available from the corresponding author upon reasonable request.

## Supplementary Materials

The following supporting information can be downloaded from https://www.mdpi.com/article/10.3390/cancers17243913/s1. Table S1: Characteristics of patients who only underwent nerve-sparing procedures; Figure S1: HRQOL domain subscales scores; Figure S2: HRQOL domain subscales scores; Figure S3: Comparison of HRQOL domain summary scores for EPIC between RS-RARP and C-RARP (experienced surgeons); Figure S4: Comparison of EPIC sexual summary score (only for nerve-sparing procedures) between RS-RARP and C-RARP (experienced surgeons).

## References

[1] Millin T. (1945). Retropubic prostatectomy a new extravesical technique: Report on 20 cases. Lancet.

[2] Moretti T.B.C., Magna L.A., Reis L.O. (2022). Surgical results and complications for open, laparoscopic, and robot-assisted radical prostatectomy: A reverse systematic review. Eur. Urol. Open Sci..

[3] Giberti C., Gallo F., Schenone M., Gastaldi E., Cortese P., Ninotta G., Becco D. (2017). Robotic prostatectomy versus brachytherapy for the treatment of low risk prostate cancer. Can. J. Urol..

[4] Huynh L.M., Osann K., Skarecky D., Ahlering T.E. (2018). Predictive modelling of 2-year potency outcomes using a novel 90-day erection fullness scale after robot-assisted radical prostatectomy. BJU Int..

[5] Galfano A., Ascione A., Grimaldi S., Petralia G., Strada E., Bocciardi A.M. (2010). A new anatomic approach for robot-assisted laparoscopic prostatectomy: A feasibility study for completely intrafascial surgery. Eur. Urol..

[6] Egan J., Marhamati S., Carvalho F.L., Davis M., O’Neill J., Lee H., Lynch J., Hankins R., Hu J., Kowalczyk K.J. (2021). Retzius-sparing robot-assisted radical prostatectomy leads to durable improvement in urinary function and quality of life versus standard robot-assisted radical prostatectomy without compromise on oncologic efficacy: Single-surgeon series and step-by-step guide. Eur. Urol..

[7] Clarebrough E.E., Challacombe B.J., Briggs C., Namdarian B., Weston R., Murphy D.G., Costello A.J. (2011). Cadaveric analysis of periprostatic nerve distribution: An anatomical basis for high anterior release during radical prostatectomy?. J. Urol..

[8] Ganzer R., Blana A., Gaumann A., Stolzenburg J.U., Rabenalt R., Bach T., Wieland W., Denzinger S. (2008). Topographical anatomy of periprostatic and capsular nerves: Quantification and computerised planimetry. Eur. Urol..

[9] Wei J.T., Dunn R.L., Litwin M.S., Sandier H.M., Sanda M.G. (2000). Development and validation of the expanded prostate cancer index composite (EPIC) for comprehensive assessment of health related quality of life in men with prostate cancer. Urology.

[10] Tokas T., Mavridis C., Bouchalakis A., Nakou C.M., Mamoulakis C. (2025). Learning Curves in Robotic Urological Oncological Surgery: Has Anything Changed During the Last Five Years?. Cancers.

[11] Ou Y.C., Yang C.R., Wang J., Cheng C.L., Patel V.R. (2008). Robotic-assisted radical prostatectomy by a single surgeon in Taiwan: Experience with the initial 30 cases. J. Robot. Surg..

[12] Eastham J.A., Kuroiwa K., Ohori M., Serio A.M., Gorbonos A., Maru N., Vickers A.J., Slawin K.M., Wheeler T.M., Reuter V.E. (2007). Prognostic significance of location of positive margins in radical prostatectomy specimens. Urology.

[13] Thompson I., Thrasher J.B., Aus G., Burnett A.L., Canby-Hagino E.D., Cookson M.S., D’Amico A.D., Dmochowski R.R., Eton D.T., Forman J.D. (2007). Guideline for the management of clinically localized prostate cancer: 2007 update. J. Urol..

[14] Galfano A., Di Trapani D., Sozzi F., Strada E., Petralia G., Bramerio M., Ascione A., Bocciardi A.M. (2013). Beyond the learning curve of the Retziussparing approach for robot-assisted laparoscopic radical prostatectomy: Oncologic and functional results of the first 200 patients with ≥1 year of follow-up. Eur. Urol..

[15] Williams S.B., Morales B.E., Huynh L.M., Osann K., Skarecky D.W., Ahlering T.E. (2017). Analysis of accessory pudendal artery transection on erections during robot-assisted radical prostatectomy. J. Endourol..

[16] Secin F.P., Touijer K., Mulhall J., Guillonneau B. (2007). Anatomy and preservation of accessory pudendal arteries in laparoscopic radical prostatectomy. Eur. Urol..

[17] Herranz Amo F. (2004). Ultrasound morphology of prostatic apex: Implications for its dissection in prostatectomy. Actas Urol. Esp..

[18] Wang Y., Cheng X., Xiong Q., Cheng S. (2023). The progress of dorsal vascular complex control strategy in radical prostatectomy. J. Int. Med. Res..

[19] Alsaid B., Bessede T., Diallo D., Moszkowicz D., Karam I., Benoit G., Droupy S. (2011). Division of autonomic nerves within the neurovascular bundles distally into corpora cavernosa and corpus spongiosum components: Immunohistochemical confirmation with three-dimensional reconstruction. Eur. Urol..

[20] Dalela D., Jeong W., Prasad M.A., Sood A., Abdollah F., Diaz M., Karabon P., Sammon J., Jamil M., Baize B. (2017). A pragmatic randomized controlled trial examining the impact of the Retzius-sparing approach on early urinary continence recovery after robot-assisted radical prostatectomy. Eur. Urol..

[21] Yu Y., Reiter R.E., Zhang M. (2025). Surgical techniques for enhancing postoperative urinary continence in robot-assisted radical prostatectomy: A comprehensive review. Int. J. Surg..

[22] Kadono Y., Nohara T., Kawaguchi S., Naito R., Kadomoto S., Iwamoto H., Yaegashi H., Shigehara K., Izumi K., Yoshida K. (2023). Contribution of Retzius-sparing robot-assisted radical prostatectomy to the mechanism of urinary continence as demonstrated by dynamic MRI. Sci. Rep..

[23] Gong W., Yan J., Cui Y., Zhang D., Ma Y. (2025). Comparison of efficacy of Retzius-sparing radical prostatectomy versus standard radical prostatectomy in the treatment of prostate cancer: A systematic review and meta-analysis. Front. Oncol..

[24] Checcucci E., Veccia A., Fiori C., Amparore D., Manfredi M., Dio M., Morra I., Galfano A., Autorino R., Bocciardi A. (2020). Retzius-Sparing Robot-Assisted Radical Prostatectomy vs the Standard Approach: A Systematic Review and Analysis of Comparative Outcomes. BJU Int..

[25] Tai T.E., Wu C.C., Kang Y.N., Wu J.C. (2020). Effects of Retzius Sparing on Robot-Assisted Laparoscopic Prostatectomy: A Systematic Review with Meta-Analysis. Surg. Endosc..

[26] Phukan C., Mclean A., Nambiar A., Mukherjee A., Somani B., Krishnamoorthy R., Sridhar A., Rajan P., Sooriakumaran P., Rai B.P. (2020). Retzius Sparing Robotic Assisted Radical Prostatectomy vs. Conventional Robotic Assisted Radical Prostatectomy: A Systematic Review and Meta-Analysis. World J. Urol..

[27] Wadhwa H., Terris M.K., Aronson W.J., Kane C.J., Amling C.L., Cooperberg M.R., Freedland S.J., Abern M.R. (2016). Long-term oncological outcomes of apical positive surgical margins at radical prostatectomy in the Shared Equal Access Regional Cancer Hospital cohort. Prostate Cancer Prostatic Dis..

[28] Taniguchi H., Kinoshita H., Koito Y., Yanishi M., Taguchi M., Mishima T., Yoshida K., Komai Y., Yasuda K., Watanabe M. (2017). Preoperative sexual status of Japanese localized prostate cancer patients: Comparison of sexual activity and EPIC scores. Aging Male.

[29] Braun M., Wassmer G., Klotz T., Reifenrath B., Mathers M., Engelmann U. (2000). Epidemiology of erectile dysfunction: Results of the ‘Cologne Male Survey’. Int. J. Impot. Res..

[30] Namiki S., Kwan L., Kagawa-Singer M., Saito S., Terai A., Satoh T., Baba S., Arai Y., Litwin M.S. (2008). Sexual function reported by Japanese and American men. J. Urol..

