# Supplementary material for: A Comparative Analysis of the Functional Outcomes Between Retzius-Sparing and Conventional Robot-Assisted Radical Prostatectomy Using the Expanded Prostate Cancer Index Composite
Source: Cancers (Basel). 2025 Dec 7;17(24):3913. doi: 10.3390/cancers17243913 (PMC12730361; doi:10.3390/cancers17243913)

TableS1. Characteristics of patients who only underwent nerve-sparing procedures

Variables	RS-RARP (n=20)	C-RARP (n=33)	p
Age (Median ± SD)	68 ± 6.8	65 ± 5.2	0.05
BMI (kg/m^2) (Median ± SD)	22.5 ± 2.8	24.9 ± 2.9	0.003
PSA (ng/ml) (Median ± SD)	7.7 ± 7.4	8.5 ± 7.9	0.74
Gleason score, n (%)			
6	4	10	0.62
7	9	16	
8	5	4	
≥9	2	3	
Clinical T stage, n (%)			
≤T2	18 (90)	31 (94)	0.63
T3	2 (10)	2 (6)	
prostate volume (ml), Median (IQR)	32.4 (22.2-38.5)	40.8 (24.4-55.6)	0.05
Nerve-sparing procedure, n (%)			
Unilateral	17 (85)	28 (85)	1.0
Bilateral	3 (15)	5 (15)	
Antihypertensive medication			
Yes	6 (30)	17 (52)	0.13
No	14 (70)	16 (48)	
Console time (minutes) (Median ± SD)	188 ± 55.6	175 ± 70.8	0.75
Blood loss (ml) (Median ± SD)	200 ± 295	250 ± 291	0.56

Abbreviations: SD, standard deviation; IQR, interquartile range; RARP, robot assisted radical prostatectomy; RS, Retzius sparing; C, conventional; BMI, body mass index; PSA, prostate specific antigen

FigureS1: HRQOL domain subscales scores.

(a)bowel function

(b)bowel bother

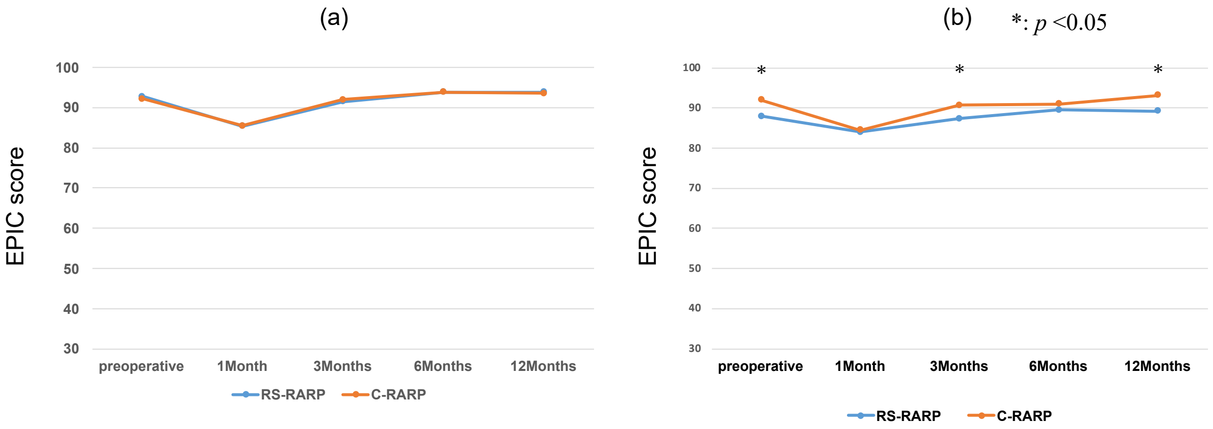

FigureS2: HRQOL domain subscales scores.

(a)sexual function

(b)sexual bother

(c)hormonal function

(d)hormonal bother

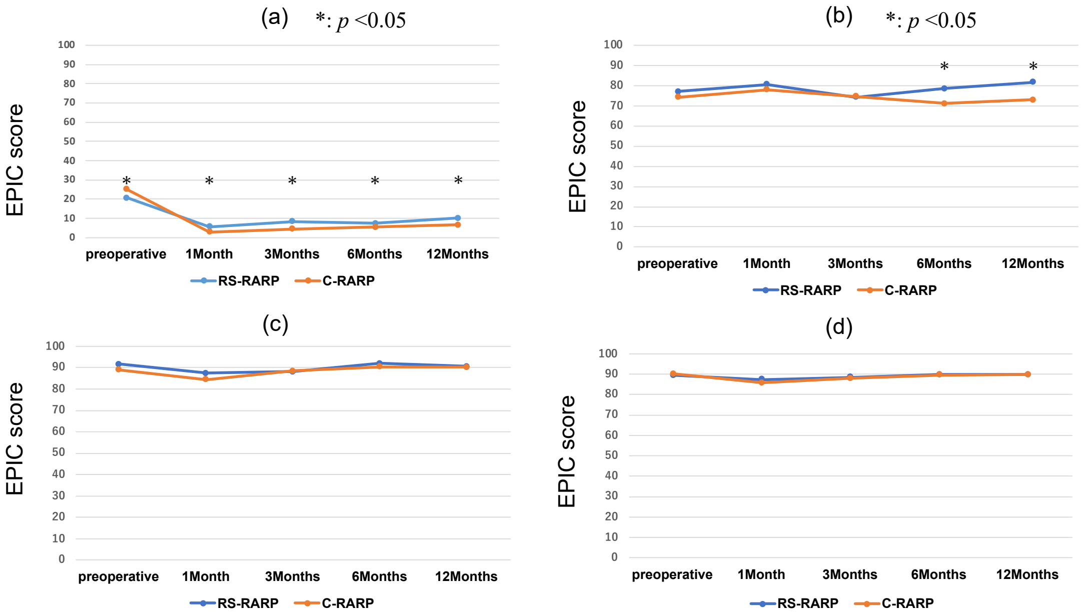

FigureS3: Comparison of HRQOL domain summary scores for EPIC between RS-RARP and C-RARP (experienced surgeons).

(a) urinary summary
(b) bowel summary
(c) sexual summary
(d) hormonal summary

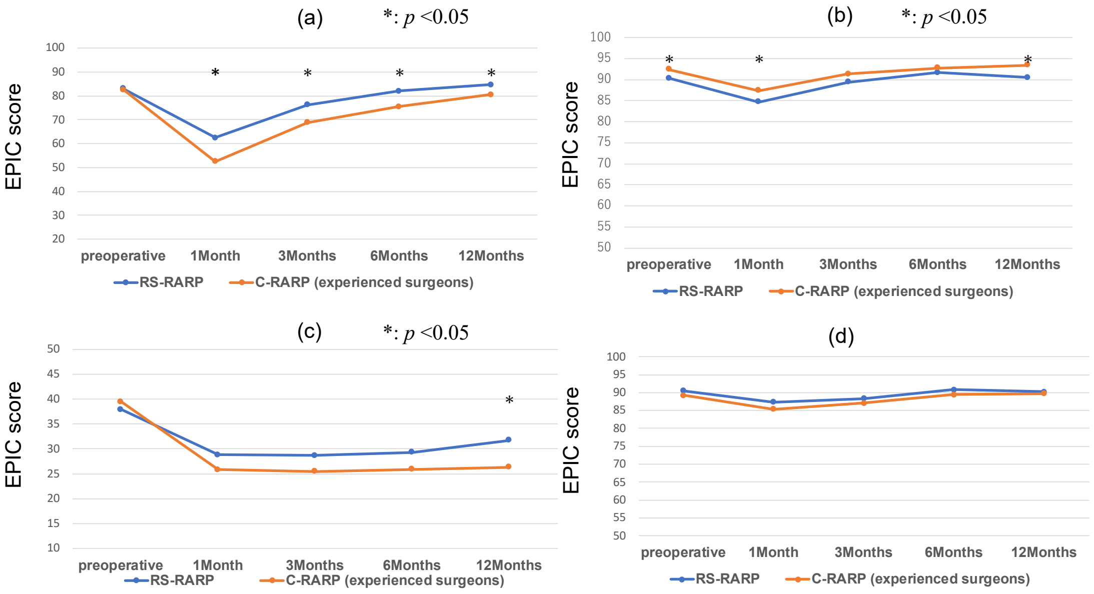

FigureS4: Comparison of EPIC sexual summary score (only for nerve-sparing procedures) between RS-RARP and C-RARP (experienced surgeons).

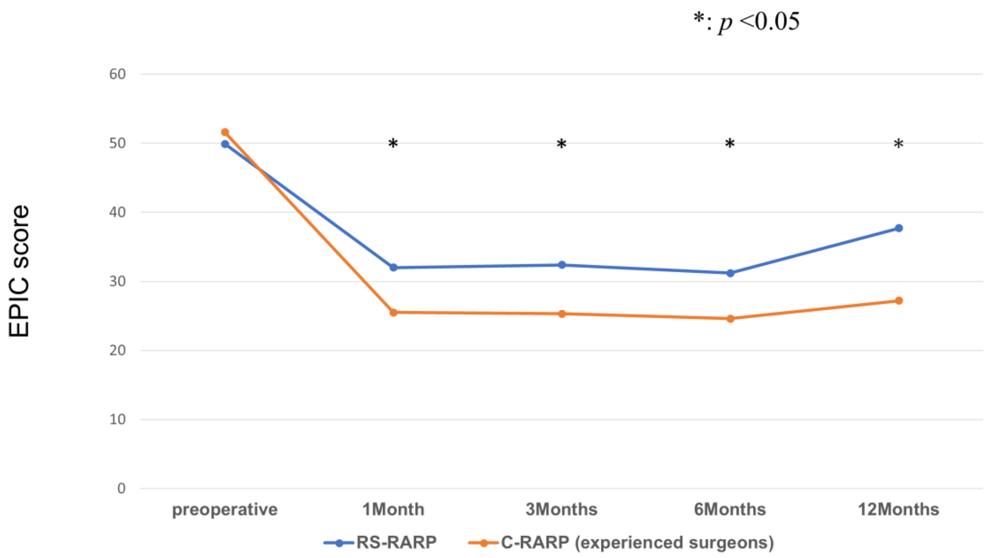

Supplement: Supplementary file 1 [file cancers-17-03913-s001.zip › cancers-4016516-supplementary.pdf]
